# Long Term Non-invasive Ventilation in Children With Central Hypoventilation

**DOI:** 10.3389/fped.2020.00288

**Published:** 2020-06-19

**Authors:** Maria Giovanna Paglietti, Irene Esposito, Manuela Goia, Elvira Rizza, Renato Cutrera, Elisabetta Bignamini

**Affiliations:** ^1^Pediatric Pulmonology & Respiratory Intermediate Care Unit, Sleep and Long-Term Ventilation Unit, Academic Department of Pediatrics, Bambino Gesù Children's Hospital, Rome, Italy; ^2^Pediatric Pulmonology & Regional Reference Centre for Pediatric Respiratory Failure and Cystic Fibrosis, Regina Margherita's Hospital, AOU Città della Salute e della Scienza, Turin, Italy

**Keywords:** central hypoventilation, arterial concentration of serum carbon dioxide, central apnea, non-invasive ventilation, children

## Abstract

Central hypoventilation (CH) is a quite rare disorder caused by some congenital or acquired conditions. It is featured by increased arterial concentration of serum carbon dioxide related to an impairment of respiratory drive. Patients affected by CH need to be treated by mechanical ventilation in order to achieve appropriate ventilation and oxygenation both in sleep and wakefulness. In fact, in severe form of Congenital Central Hypoventilation Syndrome (CCHS) hypercarbia can be present even during the day. Positive pressure ventilation via tracheostomy is the first therapeutic option in this clinical condition, especially in congenital forms. Non-Invasive ventilation is a an option that must be reserved for more stable clinical situations and that requires careful monitoring over time.

## Introduction

Central hypoventilation (CH) is a quite rare disorder caused by some congenital or acquired conditions. It is featured by increased arterial concentration of serum carbon dioxide (PaCO_2_) related to an impairment in respiratory drive (1). Diagnosis is not easy to do, as hypoventilation occurs mainly during sleep, but it must be made as early as possible, as hypoventilation can give serious long-term complications.

## Pathophysiology

Hypoventilation refers to an increased PaCO_2_ due to inadequate gas exchange. This concept is summarized in the equation PaCO_2_ = K x VCO_2_/VA where K is a constant. It reads like this: PaCO_2_ is directly proportional to the body's CO_2_ production (VCO_2_) and inversely proportional to alveolar ventilation (VA). In other words, PaCO_2_ increases when CO_2_ production increases or alveolar ventilation decreases.

The breathing system is regulated by a set of receptors sensitive to changes in partial pressure of oxygen (PaO_2_), PaCO_2_ and hydrogen potential (pH), as well as other factors, such as the stretching of bronchial smooth muscle cells. Central chemoreceptors, located bilaterally below the ventro-lateral surface of the bulb, respond to small changes in PaCO_2_. Peripheral chemoreceptors are located in structures called glomas, which are located at the bifurcation of the common carotid artery (carotid glomas), and at the aortic arch (aortic glomas) and are sensitive to changes in PaO_2_, PaCO_2_ and pH (2, 3).

In healthy subjects, PaCO_2_ is the main ventilation stimulating factor. If PaCO_2_ increases, ventilation initially increases with a corresponding greater tidal volume, followed by an increase in respiratory rate. In case of hypoxia, ventilation initially increases but then decreases over time (4).

During sleep, the resistance of the upper airways increases, muscle tone decreases (in non-REM sleep) until it is completely abolished in sleep with rapid eye movement (REM), when breathing is maintained by the diaphragm only. During non-REM sleep, ventilation decreases and causes a slight increase in PaCO_2_ and a decrease in PaO_2_ compared to wakefulness. In the REM phase, breathing is superficial and irregular and the respiratory response of the respiratory centers to O_2_ and CO_2_ is reduced (5, 6).

## Clinical Aspects

CH can be due to congenital or acquired conditions and the onset of symptoms can occur at different times depending on the underlying pathology. It's hard to find specific signs or symptoms diagnostic of central sleep apnea (CSA) and even more of hypoventilation, so clinicians must be aware that this pathway can occur in the following diseases.

**Congenital central hypoventilation syndrome (CCHS)**, also known as Ondine's curse, is a rare condition that causes primary alveolar hypoventilation, firstly described in 1970 (7). In 2003 Amiel and collaborators (8) discovered the gene responsible of the disease, which is the paired-like homeobox 2B (PHOX2B), located on chromosome 4p12. This gene is a transcription factor which plays a central role in the differentiation of the neural lineage of autonomic nervous system. Most mutations occur de novo in CCHS patients, but inheritance can derive by parents with an autosomic dominant pattern with incomplete penetrance or with a mosaicism. Majority (90%) of patients with this congenital condition have a polyalanine expansion mutations (PARMs) in exon 3 (9). Other patients present nonsense, missense, frameshift or stop codon mutations in exon 1, 2, and 3, defined as non-polyalanine expansion mutations (NPARMs) (10). Over time, knowledge of the gene let us understand that PHOX2B mutation determines the phenotype of the patients (11, 12) and must guide care choices.

Patients with CCSH often present apnea and cyanosis in neonatal period, edema and signs of right heart failure associated with pulmonary hypertension, tachycardia and sweating during sleep, early breath holding spells, episodes of unexplained convulsions, severe Apparent Life-Threatening Events (13).

CCHS is also related to some tumors of neural crest origin (ganglioneuroma, neuroblastoma, ganglioneuroblastoma), or symptoms attributed to abnormal development of neural crest cells such as the following manifestations: ophtalmologic (anisocoria, strabismus), cardiovascular (alterations of cardiac rhythm or blood pressure disregulation), endocrinologic (hyperinsulinism, hypoglycemia, hyperglycemia), gastrointestinal (constipation, Hirschprung's disease) (14).

Pathophysiology of hypoventilation in CCHS is not clear yet. Breathing and autonomic dysregulation can be related to alterations of the brain, showed by functional or structural MRI, but it is not well understood if they can be determined in neurogenesis consequent to genetic mutations or secondary to hypoxic, hypercarbic, or perfusion damage (15). Moreover there are multiple impaired processes which can be causative factors in the onset of respiratory symptoms.

**Rapid-onset obesity with hypothalamic dysfunction, hypoventilation and autonomic dysregulation (ROHHAD)** is a rare disorder presenting in childhood with rapid weight gain, hypothalamic endocrine dysfunction, and severe hypoventilation. Clinical features include ophthalmologic abnormalities, altered thermoregulation, gastrointestinal dysmotility, behavior disorders, altered pain perception and tumor of neural crest origin (16). Respiratory phenotype of ROHHAD may initially present with OSA and only develop central hypoventilation later (17). Despite many clinical aspects in common with CCHS, mutations of PHOX2B are lacking in ROHHAD patients (18). A recent paper support the hypothesis of a possible aberrant immune process in pathogenesis of the disease (19).

**Prader-Willi syndrome (PWS)** is a genetic disorder due to loss of function of specific genes in chromosome 15. It is characterized by poor muscle tone often associated to feeding problems during infancy; in childhood an insatiable appetite often leads to obesity; patients are typically affected by mild to moderate intellectual impairment, behavioral problems, short stature, hypogonadism with genital hypoplasia, incomplete pubertal development (20).

PWS patients present impairments in ventilatory control: absent or altered hypoxic ventilatory response, reduced hypercapnic ventilatory response in obese subjects, altered pulmonary mechanics due to hypotonia, respiratory muscle weakness, kyphoscoliosis, and obesity. The phenotype of sleep disordered breathing evolves over time from a central pattern in infants to an obstructive one in older children and often hesitates in excessive daytime sleepiness (21). Long-term treatment with growth hormone is indicated for children with PWS but it may determine worsening of sleep-disordered breathing soon after the initiation; therefore an evaluation by polysomnography within the first 3–6 months of starting therapy should be repeated (22).

Other congenital diseases, as Familial dysautonomia (23), Arnold Chiari malformations (24), achondroplasia (25), disorders affecting mitochondrial metabolism (26) may result in central hypoventilation, within a wider spectrum of sleep disordered breathing. These patients must be investigated as well as the increasing population with **acquired central hypoventilation** which is constituted by those previously healthy children presenting damage of respiratory centers in the brain. Gangliogliomas and consequences from neurosurgical procedures were found mostly represented (27), but central nervous system infections, encephalitis, trauma, and other central nervous system tumors can be other onset reasons.

## Diagnosis

Hypoventilation is caused by insufficient alveolar ventilation which causes altered blood gas values. American Academy of Sleep Medicine (AASM) (28, 29) scored hypoventilation during sleep when >25% of the total sleep time as measured by either the arterial PCO_2_ or surrogate is spent with a PCO_2_ > 50 mmHg. The process of arterial blood is the gold standard method to diagnose hypoventilation but its use during sleep is difficult and normally not available in a sleep laboratory. Therefore, AASM stated that an elevated PaCO_2_ obtained immediately after waking allows diagnosis of hypoventilation during sleep. So, surrogate measures such as transcutaneous PCO_2_ (PtcCO_2_) and end-tidal PCO_2_ (PETCO_2_) are commonly used and considered acceptable methods for assessing pediatric alveolar hypoventilation.

PtcCO_2_ has a good correlation with PaCO_2_, but its response time to acute changes in ventilation is longer than the PETCO_2_. The American Association for Respiratory Care Clinical Practice Guidelines (30) recommend that arterial blood gas values be compared to transcutaneous readings, in order to verify them in acute situations. PtcCO_2_ is a very useful device in order to monitor the adequacy of ventilation (31).

Monitoring of exhaled CO_2_ (capnography) is very used in children, but it is not accurate with low tidal volume and fast respiratory rates, as in infants or in acute distress, because a defined plateau in expiratory curve is not available. Usually PaCO_2_ is higher of PETCO_2_ between 2 and 7 mmHg (28). End-tidal PCO_2_ monitoring cannot be used during application of supplemental oxygen or during mask ventilation because exhaled gas sample is diluted and in case of mouth breathing.

Polysomnography is the gold standard to score central, obstructive or mixed events. Usually apneas recur during REM sleep, but in CCHS the events occur during NREM one. Central apnea is defined by cessation of airflow without respiratory effort and duration of 20 s or longer, or duration of two breaths during baseline breathing and association with an arousal or ≥3% oxygen desaturation, or, for infants younger than 1 year of age, duration of two breaths during baseline breathing and association with a decrease in heart rate to <50 beats per minute for at least 5 s or <60 beats per minute for 15 s (29).

## Management

Clinicians must be aware of the different therapeutic options and be able to choose the correct one, even because the amount of ventilator assistance required in these syndromes is extremely variable.

### Ventilation

In patients requiring continuous ventilation, positive-pressure ventilation via tracheostomy is the most common and effective method of treatment (1). American Thoracic Society statement still advice this ventilation option in the first years of life for CCHS patients (13) since ventilator system becomes more stable with age.

If possible, tracheostomy tube should be cuffless and smaller than airways dimension, in order to reduce the risk of tracheomalacia and to consent the utilization of speaking-valve when patient is not ventilated.

Non-invasive positive-pressure ventilation (NPPV) provides adequate ventilation through a mask; this modality can be chosen in patients requiring ventilation only during sleep. Its major benefit is the avoidance of tracheostomy and its complications. Nasal masks are the most used, as in all populations that perform long-term ventilation. Bilevel positive airway pressure (BiPAP) ventilation mode is the most used as it provides variable continuous flow, with fixed inspiratory and expiratory positive airway pressure whose difference is proportional to tidal volume. As children with central hypoventilation syndromes don't trigger the ventilator adequately during sleep, the timed mode with a set respiratory rate is to be preferred.

Recently intelligent volume-assured pressure support (iVAPS), which can modulate pressure support to ensure constant alveolar ventilation (32) has been proponed for CCHS patients. The rationale for the proposal is that, since these patients have variable ventilation between REM and NREM sleep, pressure is automatically modulated to better control carbon dioxide levels throughout the night (33). Some manufacturers indicate that iVAPS is FDA cleared for patients weighing more than 66 lb (>30 kg). A 10 months old CCHS infant report shows that, in case of availability of an adequate nasal mask, of a correct education of the parents and of a mid-face hypoplasia prevention program, it is possible to use NIV in AVAPS mode (average volume assured pressure support), which it is similar to iVAPS, even in younger children. This ensures less oscillation of the PaCO_2_ values during sleep (34).

Mid-face hypoplasia is a frequent complication of mask ventilation, especially if introduced in infancy. Provide two different masks with different points of pressure on the face is a good strategy, but also a closely follow up by pediatric maxillofacial surgeon and orthodontist should be performed.

An increasing number of children with CCHS have been successfully transitioned from invasive ventilation to BiPAP ventilation and recently a proposal of an algorithm for decannulation was published (35). By the moment there are not specific indications about optimal time to switch from tracheostomy to NPPV because it is a choice closely linked to the patient and his family, but ending the decannulation program before adolescence can be a good option.

Negative-pressure ventilation (NPV) causes inspiration as it creates a negative inspiratory pressure around the chest of the patient, using a bell of his appropriate size.

A small group of CCHS children passed from invasive ventilation to negative pressure ventilation removing tracheostomy (36) but its use is really limited because of non-portability, obliged supine position during sleep, skin irritation, uncomfort, risk of obstructive sleep apneas relied to asynchrony between vocal cords opening and thoracic inspiratory efforts (1).

In patients with central hypoventilation syndromes, a second ventilator in the home is necessary, in the event of technical failure. Moreover, if ventilation is continuous, a power generator or a continuous energy supply according with local energy company must be available.

### Diaphragm Pacing

Diaphragm pacing can generate breathing using the child's own diaphragmatic contraction by the electrical stimulation of the phrenic nerve (37). This device is very useful in patients with central hypoventilation syndrome requiring ventilator support 24 h a day. Contrary to adults, in children ventilation is still necessary as diaphragm needs a rest some hours a day but, moreover, because complete airway obstruction after pacing implantation is described as upper airway muscle contraction is not synchronous with paced inspiration (38).

**Figure 1:**
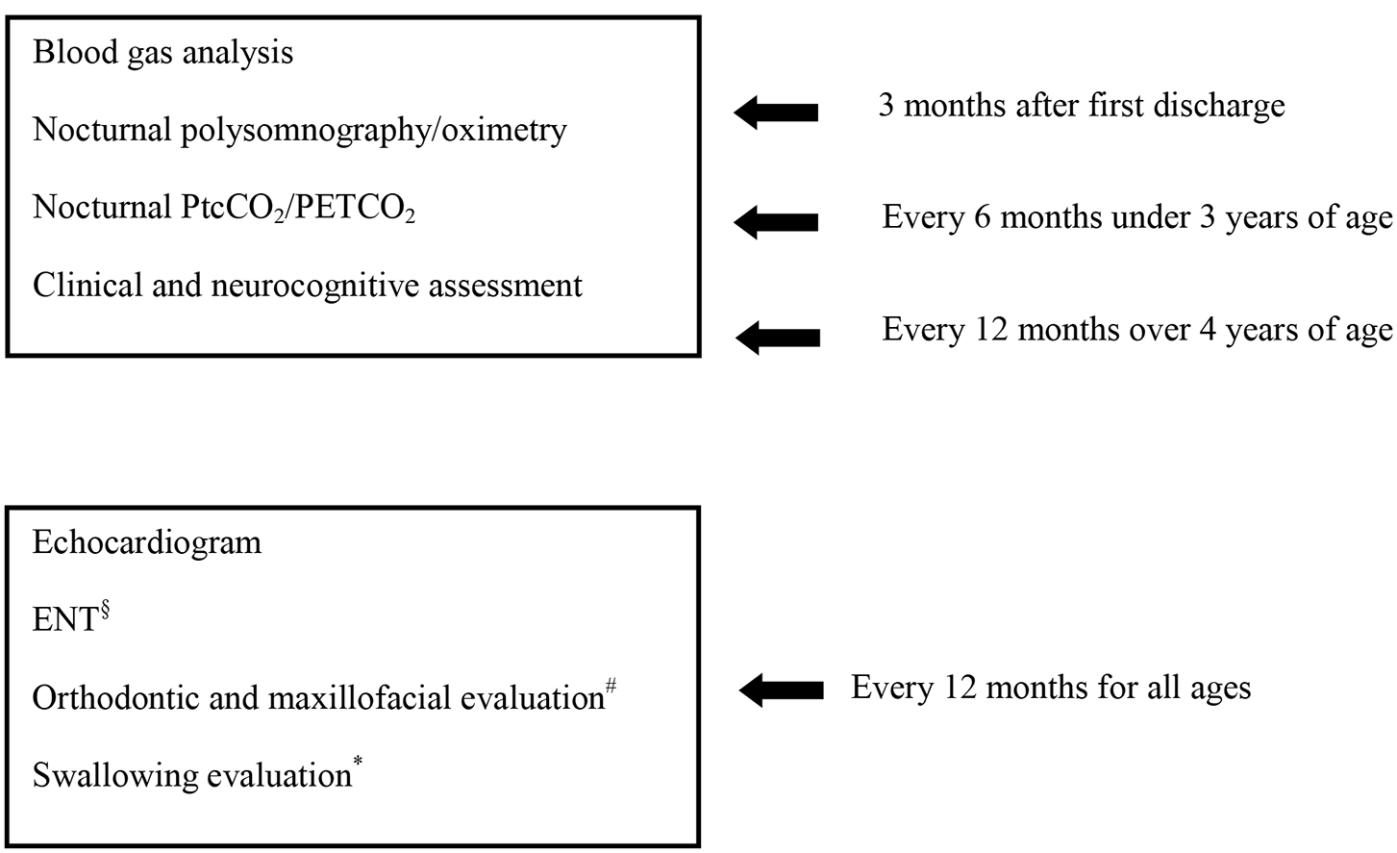
Follow up program for clinically stable patients with central hypoventilation. ^§^In case of tracheostomy. ^#^In case of NIV, after 3 years of age. ^*^Evaluation carried out as needed.

These patients require careful and structured management over time. In Figure 1 we suggested the tests to be performed over time for central hypoventilation.

Obviously, the pathologies underlying hypoventilation will require specific follow up for the disease and its complications.

Last but not least, the follow up must be individualized on the basis of the specific characteristics of the patient, with a prompt response in the event of exacerbations or clinical worsening.

## Conclusion

Central hypoventilation syndrome is the clinical manifestation of a heterogeneous group of pathologies. Therefore, clinician must adequately treat respiratory impairment, but also other aspects related to primary disease with a multidisciplinary approach.

Ventilation must be suited individually, basing on clinical pathway, on the amount of daily time spent in ventilation, on patient and family decision, if possible. Non-invasive ventilation has multiple advantages, but it requires a considerable effort and needs a careful monitoring over time.

## Author Contributions

MP designed the review in collaboration with IE. MG and ER contributed to the content. EB and RC revised the content of review.

## Conflict of Interest

The authors declare that the research was conducted in the absence of any commercial or financial relationships that could be construed as a potential conflict of interest.
